# Synthesis and structure of *catena*-poly[[[bis­(pyridin-2-yl)amine]­cadmium(II)]-di-μ_2_-azido]

**DOI:** 10.1107/S2056989026000289

**Published:** 2026-01-16

**Authors:** Zouaoui Setifi, Fatima Setifi, Joel T. Mague, Mohammed Hadi Al-Douh

**Affiliations:** a Département de Technologie Faculté de Technologie Université 20 Août 1955-Skikda BP 26 Route d'El-Hadaiek Skikda 21000 Algeria; b Laboratoire de Chimie Ingénierie Moléculaire et Nanostructures (LCIMN) Université Ferhat Abbas Sétif 1 Sétif 19000 Algeria; c Department of Chemistry, Tulane University, New Orleans, LA 70118, USA; d Chemistry Department, Faculty of Science, Hadhramout University, Mukalla, Hadhramout, Yemen; University of Aberdeen, United Kingdom

**Keywords:** solvothermal synthesis, crystal structure, coordination polymer, cadmium, azide, amine

## Abstract

In the title compound, [Cd(N_3_)_2_(C_10_H_9_N_3_)]_*n*_, the cadmium(II) ion displays a distorted CdN_6_ octa­hedral geometry arising from one bidentate ligand and four azide ions and forms zigzag polymeric [100] chains *via* the bridging azide ions, both of which show μ_1,1_ (end-on) coordination. Adjacent chains are linked into layers *via* N—H⋯N hydrogen bonds. Hirshfeld surface analysis was used to qu­antify the inter­molecular inter­actions.

## Abstract 2

The title compound, [Cd(N_3_)_2_(C_10_H_9_N_3_)]_*n*_, was prepared solvothermally and characterized crystallographically. In the crystal, adjacent polymeric chains are linked into layers *via* N—H⋯N hydrogen bonds.

## 1. Chemical context

Cadmium(II) coordination polymers containing polynitrile or pseudohalide ligands have been widely investigated because of their photoluminescence (Addala *et al.*, 2019 ▸; Majumder *et al.*, 2017 ▸) or photocatalysis (Roy *et al.*, 2017 ▸) properties. Generally, the crystal chemistry of the Cd^II^ ion is dominated by coordination numbers of four to six (Setifi *et al.*, 2017 ▸; Liu *et al.*, 2016 ▸). As for the choice of anionic ligands, pseudohalides are considered as a good linker species. In particular, the azide ligand is an attractive bridging ligand due to the variability of its coordination modes, such as the common μ_1,1_ (end-on, EO) and μ_1,3_ (end-to end, EE) modes with single or double azide bridges (Setifi *et al.*, 2025 ▸; Merabet *et al.*, 2023 ▸). Therefore, such anionic ligands are used for studying magnetochemistry and for the construction of coordination frameworks (Benamara *et al.*, 2021 ▸; Merabet *et al.*, 2022 ▸).
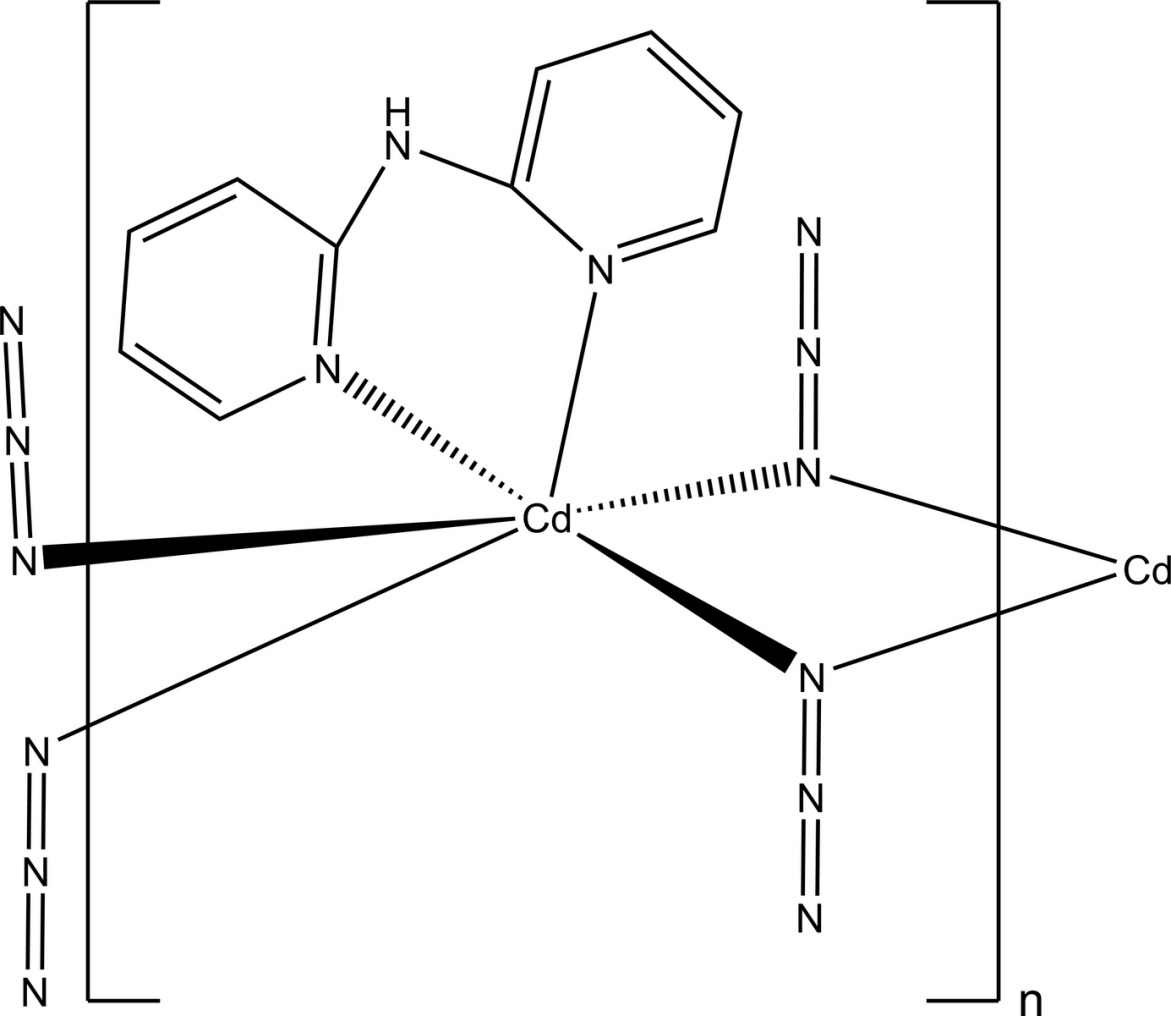


As part of our ongoing work in this area, the title one-dimensional Cd^II^ coordination polymer, [Cd(N_3_)_2_(C_10_H_9_N_3_)]_*n*_ (**I**), was synthesized and characterized and is reported herein.

## 2. Structural commentary

**Figure 1:**
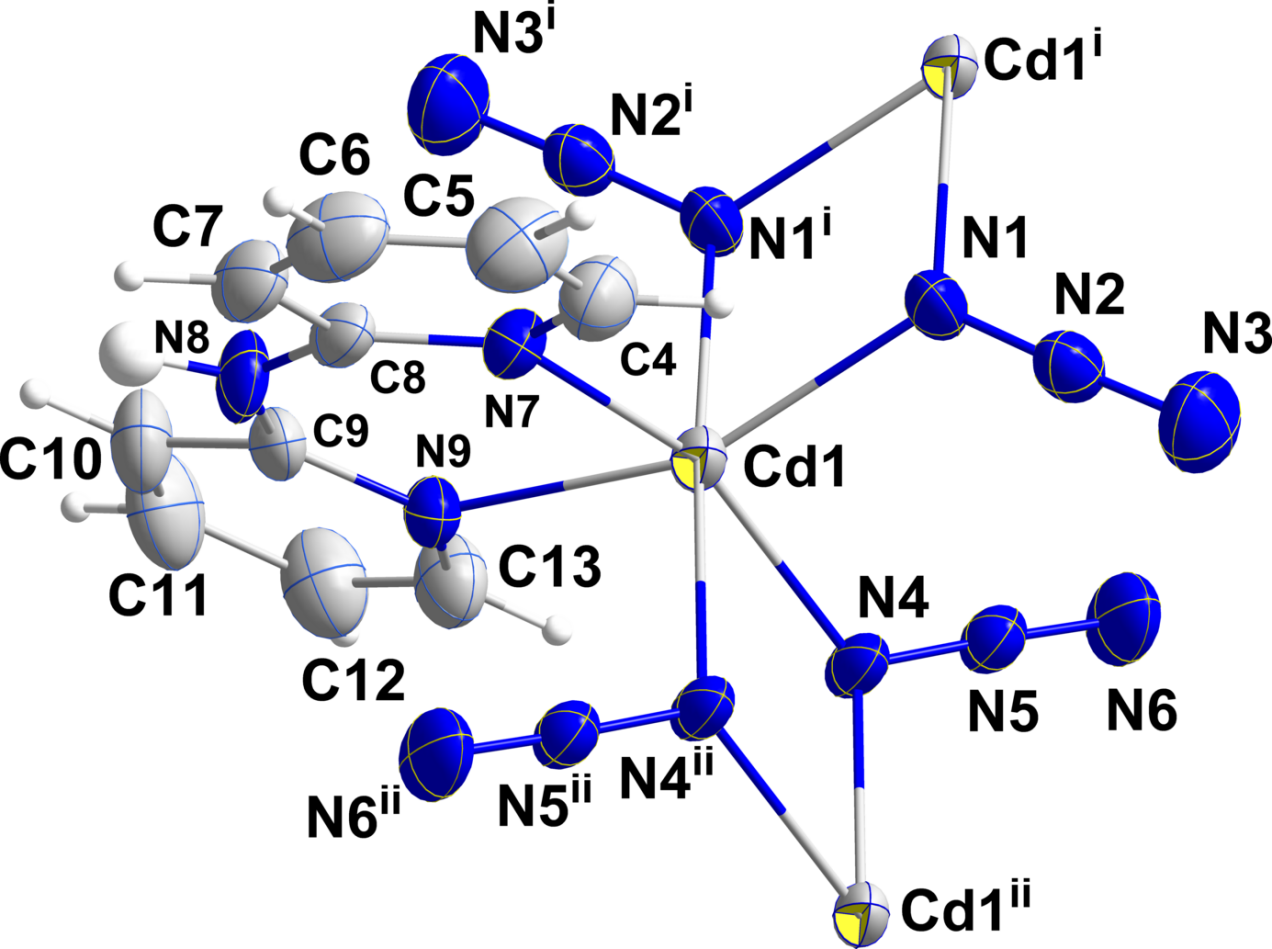
The coordination sphere of the metal ion in (**I**) with 50% probability ellipsoids. Symmetry codes: (i) −*x* + 1, −*y* + 1, −*z* + 1; (ii) −*x* + 2, −*y* + 1, −*z* + 1.

In compound (**I**), the cadmium ion adopts a distorted CdN_6_ octa­hedral coordination geometry (Table 1 ▸) provided by two N atoms from the chelating ligand (N7 and N9) in *cis* positions, two from the μ_2_;η^1^-azide ions in the asymmetric unit and the last two from symmetry generated μ_2_;η^1^-azide ions (N1^i^ at 1 − *x*, 1 − *y*, 1 − *z* and N4^ii^ at 2 − *x*, 1 − *y*, 1 − *z*) (Fig. 1 ▸). Part of the distortion results from the small bite angle of the chelating ligand giving an N7—Cd1—N9 angle of 80.59 (9)° while the N1—Cd1—N4 angle, at 95.09 (10)°, is closer to the ideal value. Four Cd—N distances are in the narrow range of 2.293 (3)–2.332 (2) Å but the other two are notably longer at 2.394 (3) Å (Cd1—N1^i^) and 2.436 (3) Å (Cd1—N4^ii^) (Fig. 1 ▸) making the Cd(μ_2_(N_3_)_2_)Cd units unsymmetrical. This also leads to two different Cd⋯Cd distances with Cd1⋯Cd1^i^ being 3.6812 (4) Å while the Cd1⋯Cd1^ii^ separation is 3.7432 (4) Å (Fig. 1 ▸).

## 3. Supra­molecular features

**Figure 2:**
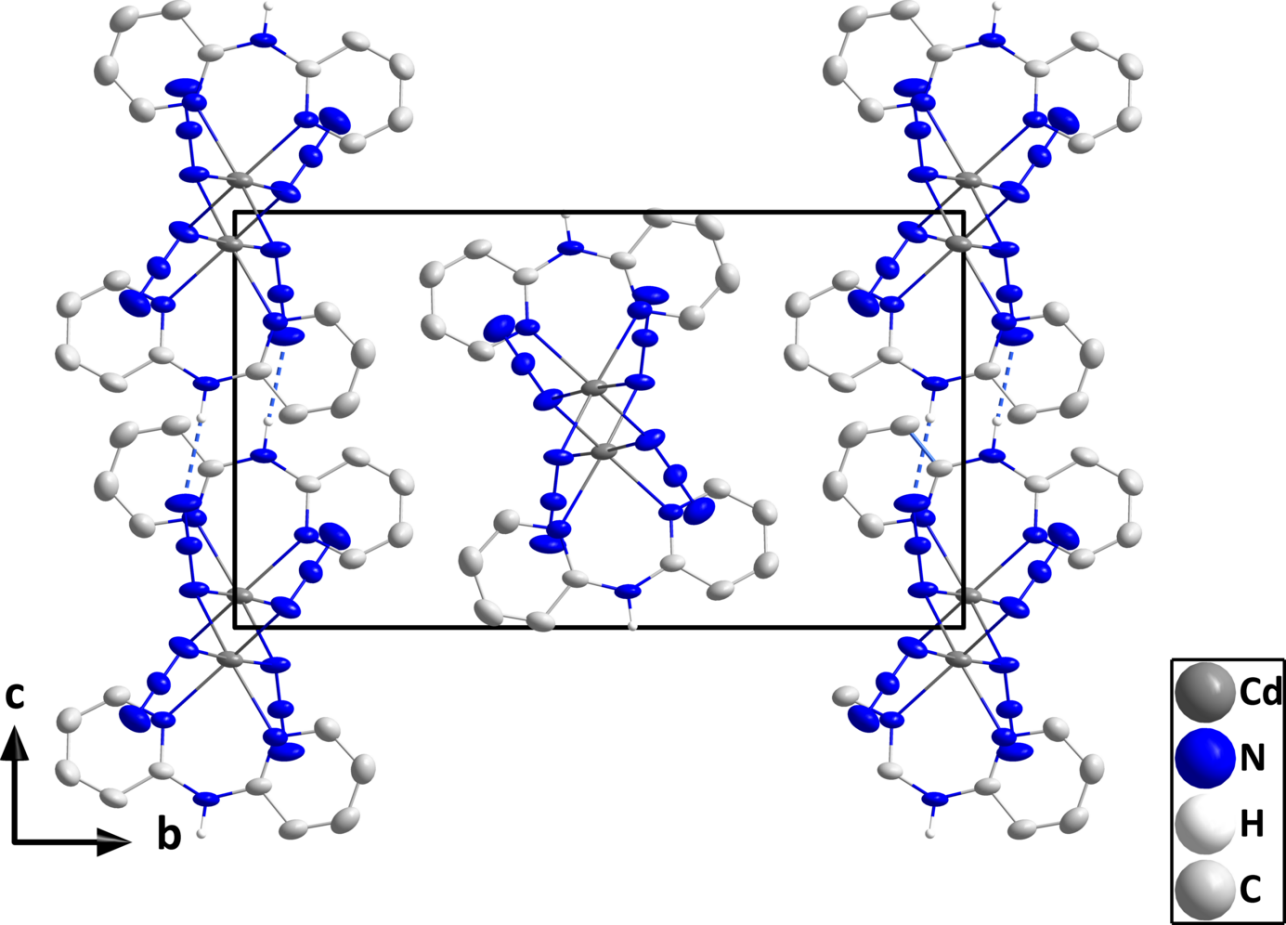
The packing in (**I**) viewed along the *a*-axis direction showing end views of several chains. The N—H⋯N hydrogen bonds are depicted by dashed lines and non-inter­acting hydrogen atoms are omitted for clarity.

In the crystal, the *cis* position of the bridging azide ligands leads to the formation of zigzag chains built up from Cd(μ_2_(N_3_)_2_)Cd units extending along the *a*-axis direction, which are connected by N8—H8⋯N6 hydrogen bonds (Table 2 ▸) into layers lying parallel to the *ac* plane (Figs. 2 ▸ and 3 ▸).

## 4. Database survey

**Figure 4:**
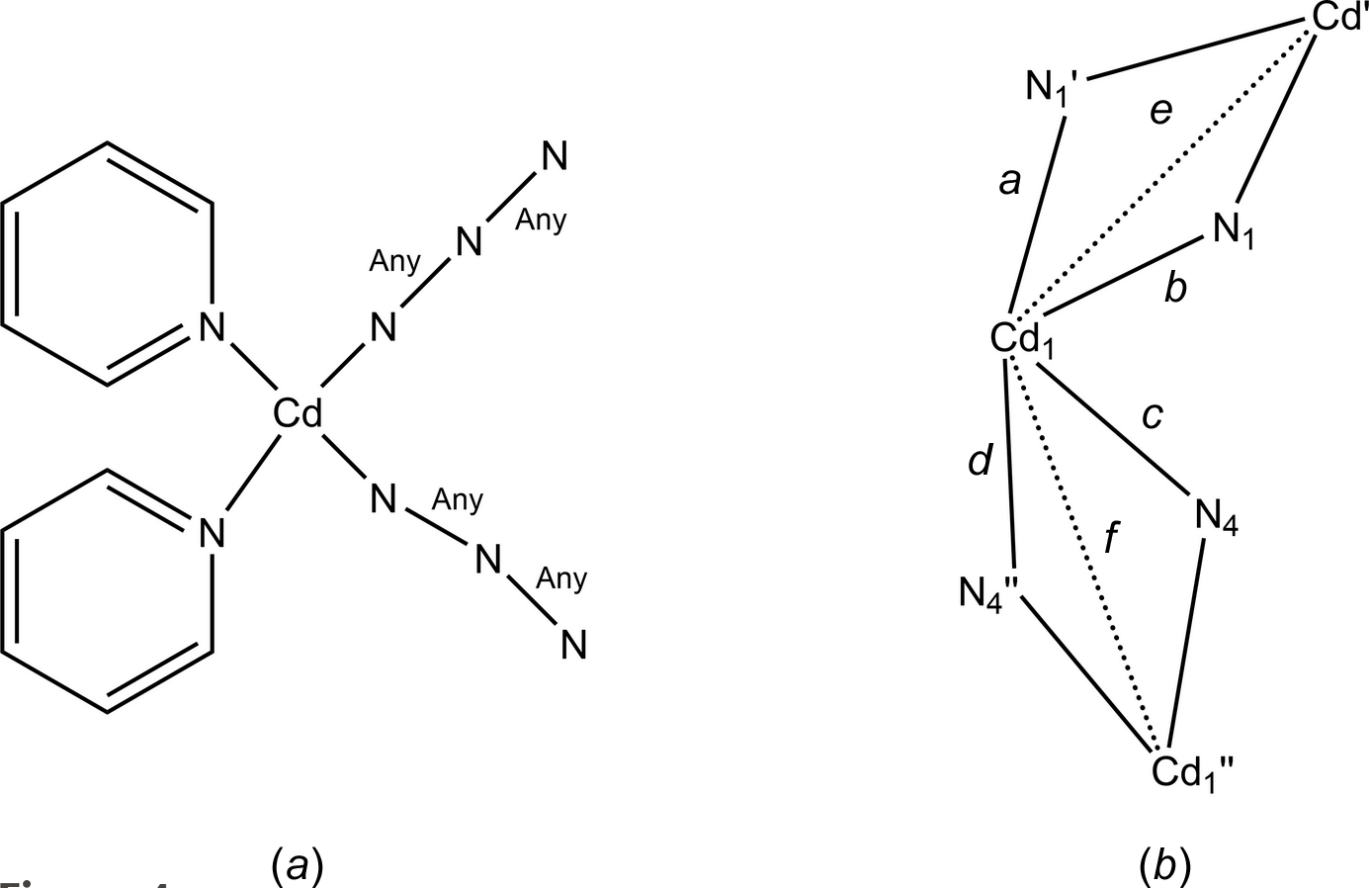
(*a*) The search fragment used where ‘Any’ refers to any bond type (single, double or delocalized) in the CSD search, and (*B*) the key for column headings in Table 3 ▸.

A search of the Cambridge Structural Database [CSD, updated to September 2025 (Groom *et al.*, 2016 ▸)] with the search fragment shown in Fig. 4 ▸*a* gave 65 hits, 21 of which contained coordinated azide ions. Of these, 16 were considered most similar to the title compound while one was monomeric and the remaining four contained exclusively Cd—N=N=N—Cd bridging units. Table 3 ▸ lists the title compound and the most similar ones with pertinent geometric details. The compounds with refcodes FEBKED to UMUSUS contain a chelating ligand similar to that in the title compound so that the two Cd(μ_2_(N_3_)_2_)Cd units are *cis* to one another and unsymmetrically bridged as well. The Cd—N distances are comparable although the short–long pattern is not always in the same order. Except for OWOGAK, which has two different Cd⋯Cd separations as is the case with the title mol­ecule, the Cd⋯Cd separations are equivalent by symmetry. For FARZEF, FARZIJ and TERHUT, pairs of Cd ions are bridged either by one μ_2_;η^1^-N_3_ ion and one μ_3_;η^1^-N_3_ ion or by two μ_3_;η^1^-N_3_ ions. Here, the Cd—N distances to the μ_2_;η^1^-N_3_ ion are comparable to the shorter ones seen in (**I**) but those to the μ_3_;η^1^-N_3_ grouping are noticeably longer as expected. The last group contains Cd(μ_2_(N_3_)_2_)Cd units which are *trans* to one another but the bridging units are still unsymmetrical except for GOYROD where site symmetry requires them to be symmetrical.

## 5. Hirshfeld surface analysis

**Figure 5:**
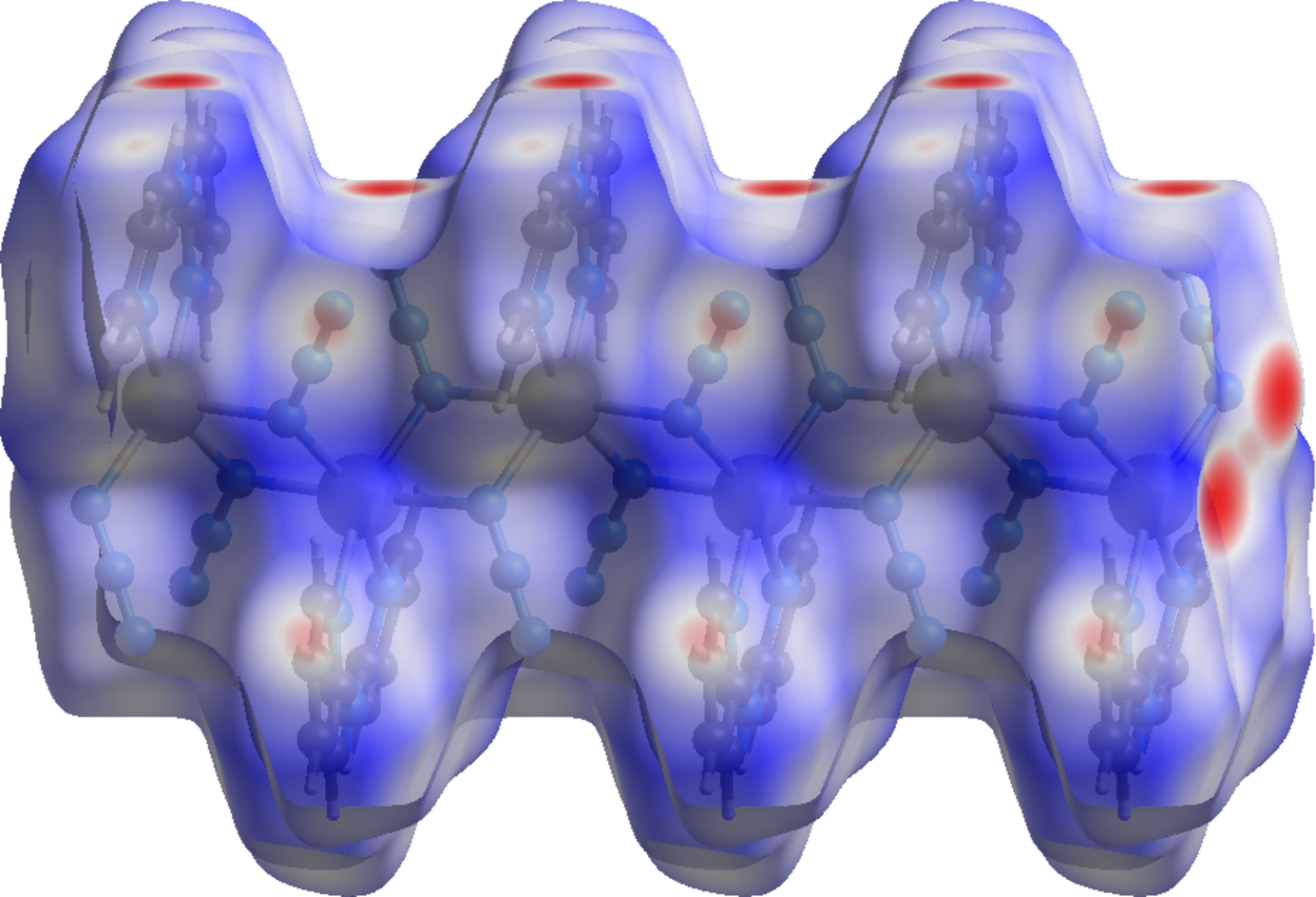
The *d*_norm_ Hirshfeld surface for (**I**).

**Figure 6:**
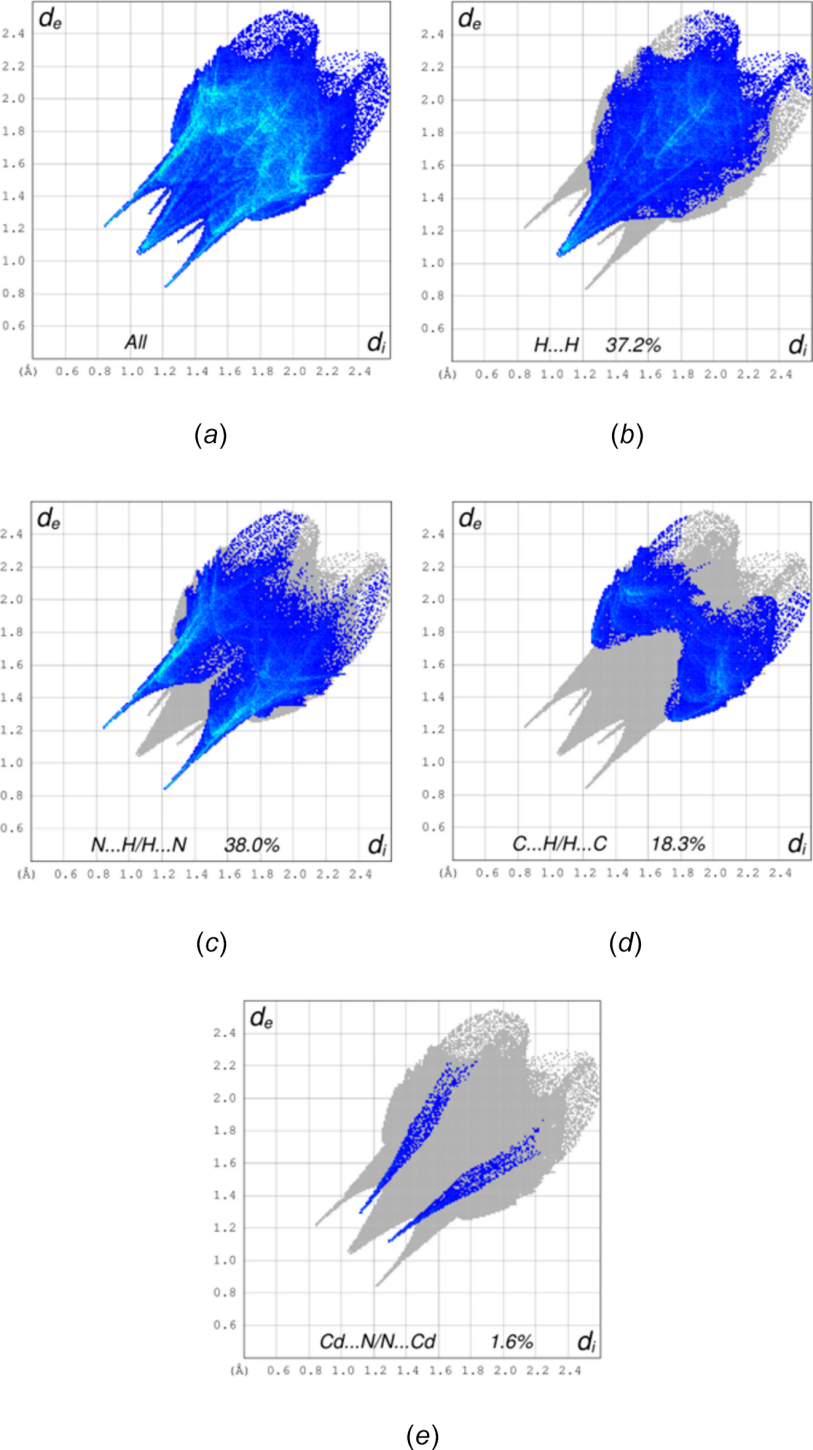
Two-dimensional fingerprint plots for (**I**) showing all inter­actions (*a*) and those delineated into H⋯H (*b*), N⋯H/H⋯N (*c*), C⋯H/H⋯C (*d*) and Cd—N (*e*) inter­actions.

A Hirshfeld surface (HS) analysis was performed using CrystalExplorer (Spackman *et al.*, 2021 ▸) to explore the inter­molecular inter­actions in the crystal of (**I**). Descriptions and inter­pretations of the plots obtained have been published (Tan *et al.*, 2019 ▸). The *d*_norm_ HS for a portion of one chain is shown in Fig. 5 ▸ with the bright red spots on the right side showing the sites of N—Cd bonds that continue the chain. The red spots on the top of the surface indicate the locations of the N—H⋯N hydrogen bonds, which connect the chains. Fig. 6 ▸ shows the two-dimensional fingerprint plots with Fig. 6 ▸*a* showing all inter­molecular inter­actions. This is characterized by two pairs of sharp peaks and a broader central one. Delineation of these into specific atom⋯atom inter­actions shows the central peak to represent H⋯H inter­actions at 37.2% of the total and the pair with tips at *d*_e_ + *d*_i_ ≃ 2.2 Å (Fig. 6 ▸*c*) consistent with the N—H⋯N hydrogen bonds at 38.0% of the total. The C⋯H/H⋯C inter­actions constitute 18.3% of the total and appear as a pair of broad peaks at *d*_e_ + *d*_i_ ≃ 3 Å (Fig. 6 ▸*d*). These do not appear to represent any *specific* inter­actions as calculations of inter­molecular distances do not show any C—H⋯π(ring) inter­actions to be present. Finally, the pair of sharp peaks with *d*_e_ + *d*_i_ ≃ 2.4 Å (Fig. 6 ▸*e*) can be attributed to the Cd—N bonds mentioned above that continue the chain beyond that fragment used in the calculation of the HS.

## 6. Synthesis and crystallization

The title compound was prepared under solvothermal conditions from a mixture of cadmium(II) nitrate tetra­hydrate (62 mg, 0.20 mmol), 2,2′-di­pyridyl­amine (17 mg, 0.10 mmol), sodium azide (26 mg, 0.40 mmol), N,N-di­methyl­formamide (10 ml) and water (7 ml), which was sonicated for 30min. Then the reaction mixture was transferred to a Teflon-lined stainless steel reactor and heated to 403 K for 2 days. After cooling to room temperature at a rate of 10 K h^−1^, colourless block-shaped crystals of (**I**) were collected.

## 7. Refinement

Crystal data, data collection and structure refinement details are summarized in Table 4 ▸. The N-bound H atom was located in a difference map and its position was freely refined. The C-bound H atoms were located geometrically (C—H = 0.93 Å) and refined as riding atoms.

## Supplementary Material

Crystal structure: contains datablock(s) I, global. DOI: 10.1107/S2056989026000289/hb8184sup1.cif

Structure factors: contains datablock(s) I. DOI: 10.1107/S2056989026000289/hb8184Isup2.hkl

CCDC reference: 2522304

Additional supporting information:  crystallographic information; 3D view; checkCIF report

## Figures and Tables

**Figure 3:**
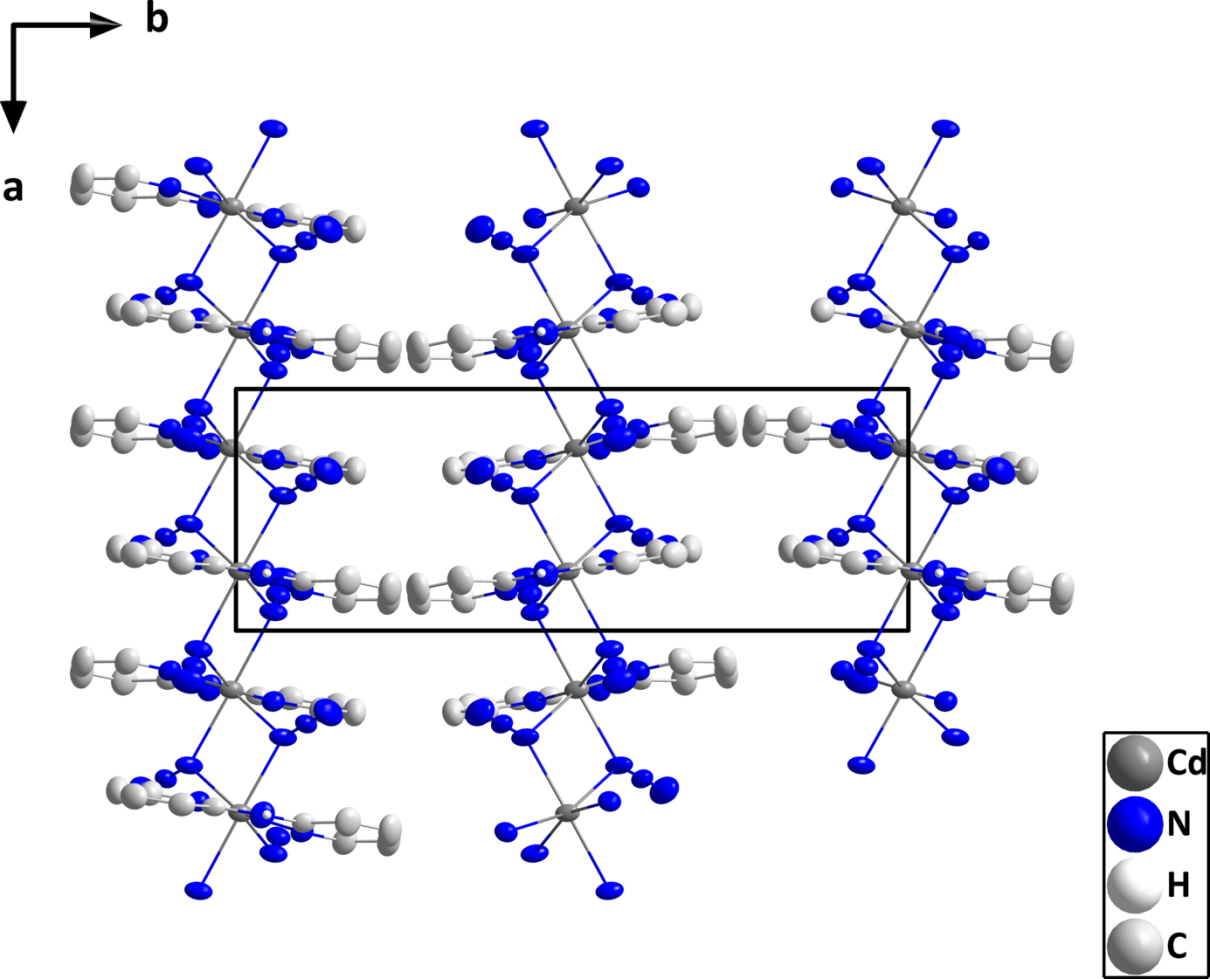
The packing in (**I**) viewed along the *c*-axis direction showing side views of several chains. The N—H⋯N hydrogen bonds are depicted by dashed lines and non-inter­acting hydrogen atoms are omitted for clarity.

**Table 1:** Selected geometric parameters (Å, °)

Cd1—N1	2.293 (3)	Cd1—N4	2.332 (2)
Cd1—N9	2.330 (2)	Cd1—N1^i^	2.394 (3)
Cd1—N7	2.330 (2)	Cd1—N4^ii^	2.436 (3)
			
N1—Cd1—N9	158.28 (9)	N7—Cd1—N1^i^	100.49 (10)
N1—Cd1—N7	94.44 (10)	N4—Cd1—N1^i^	99.89 (10)
N9—Cd1—N7	80.59 (9)	N1—Cd1—N4^ii^	102.19 (10)
N1—Cd1—N4	95.09 (10)	N9—Cd1—N4^ii^	98.22 (9)
N9—Cd1—N4	96.82 (9)	N7—Cd1—N4^ii^	83.17 (9)
N7—Cd1—N4	159.02 (9)	N4—Cd1—N4^ii^	76.57 (10)
N1—Cd1—N1^i^	76.52 (11)	N1^i^—Cd1—N4^ii^	176.17 (9)
N9—Cd1—N1^i^	83.57 (9)		

Symmetry codes: (i) 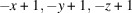; (ii) 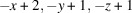.

**Table 2:** Hydrogen-bond geometry (Å, °)

*D*—H⋯*A*	*D*—H	H⋯*A*	*D*⋯*A*	*D*—H⋯*A*
N8—H8⋯N6^iii^	0.89 (1)	2.21 (1)	3.086 (4)	174 (4)

Symmetry code: (iii) 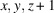.

**Table 3:** Inter­atomic distances in Cd(μ-N_3_)_2_ units

REFCODE	*a* ^1^	*b* ^1^	*c* ^1^	*d* ^1^	*e* ^1^	*f* ^1^	Reference
(**I**)	2.394 (3)	2.293 (3)	2.332 (2)	2.436 (3)	3.6812 (4)	3.7432 (4)	This work
FEBKED	2.334 (2)	2.399 (2)	2.399 (2)	2.334 (2)	307917 (9)	3.7917 (9)	He & Lu (2004 ▸)
FEBKED01	2.312 (3)	2.422 (3)	2.422 (3)	2.312 (3)	3.7728 (2)	3.7728 (2)	Abu-Youssef 2005 ▸
OWOGAK	2.445 (2)	2.300 (2)	2.2811 (19)	2.3610 (18)	3.7266 (2)	3.7871 (2)	Marandi *et al.* (2016 ▸)
QUXZOZ	2.411 (2)	2.303 (2)	2.303 (2)	2.411 (2)	3.7643 (4)	3.7728 (2)	Chen *et al.* (2010 ▸)
UMUSUS	2.367 (4)	2.322 (5)	2.323 (5)	2.367 (4)	3.6327 (9)	3.6327 (9)	Wan *et al.* (2016 ▸)
							
FARZEF	2.283 (6)	2.490 (5)^2^	2.408 (4)^2^	2.326 (6)	3.7351 (6)	3.6992 (7)	Machura *et al.* (2012 ▸)
FARZIJ	2.278 (6)	2.376 (4)^2^	2.396 (5)^2^	2.252 (5)	3.6763 (10)	3.6432 (10)	Machura *et al.* (2012 ▸)
TEPHUT	2.283 (4)	2.439 (4)^2^	2.471 (3)^2^	2.314 (4)	3.6146 (5)	3.7018 (5)	Bai *et al.* (2013 ▸)
							
GIWYER	2.371 (2)	2.351 (2)	2.351 (2)	2.371 (2)	3.6935 (11)	3.6935 (11)	Goher *et al.* (2008 ▸)
GIWYIV	2.369 (5)	2.312 (4)	2.355 (6)	2.326 (5)	3.5665 (19)	3.5516 (19)	Goher *et al.* (2008 ▸)
GOYROB	2.3441 (18)	2.3421 (17)	2.3421 (17)	2.3441 (18)	3.5298 (3)	3.5298 (3)	Mautner *et al.* (2015 ▸)
KABSUB	2.411 (3)	2.308 (2)	2.411 (3)	2.308 (2)	3.6267 (2)	3.6267 (2)	Yang *et al.* (2010 ▸)
WUBSIV	2.359 (6)	2.329 (6)	2.329 (6)	2.585 (6)	3.7050 (16)	3.9291 (16)	Goher *et al.* (2002 ▸)

Notes: (1) see Fig. 4 ▸*b* for key; (2) distance to μ_3_-N_3_ ion.

**Table 4:** Experimental details

Crystal data	
Chemical formula	[Cd(N_3_)_3_(C_10_H_9_N_3_)]
*M*_r_	367.66
Crystal system, space group	Monoclinic, *P*2_1_/*c*
Temperature (K)	298
*a*, *b*, *c* (Å)	6.6655 (5), 18.5106 (17), 10.5746 (9)
β (°)	93.612 (3)
*V* (Å^3^)	1302.13 (19)
*Z*	4
Radiation type	Mo *K*α
μ (mm^−1^)	1.68
Crystal size (mm)	0.34 × 0.21 × 0.17

Data collection	
Diffractometer	Bruker D8 Quest PHOTON 100 CCD
Absorption correction	Multi-scan (*SADABS*; Krause *et al.*, 2015 ▸)
*T*_min_, *T*_max_	0.796, 0.877
No. of measured, independent and observed [*I* > 2σ(*I*)] reflections	64159, 6322, 5357
*R*_int_	0.050
(sin θ/λ)_max_ (Å^−1^)	0.835

Refinement	
*R*[*F*^2^ > 2σ(*F*^2^)], *wR*(*F*^2^), *S*	0.055, 0.096, 1.37
No. of reflections	6322
No. of parameters	185
No. of restraints	1
H-atom treatment	H atoms treated by a mixture of independent and constrained refinement
Δρ_max_, Δρ_min_ (e Å^−3^)	1.19, −1.10

Computer programs: *APEX4* and *SAINT* (Bruker, 2018 ▸), *SHELXT2014/5* (Sheldrick, 2015*a* ▸), *SHELXL2019/3* (Sheldrick, 2015*b* ▸), *DIAMOND* (Brandenburg & Putz, 2012 ▸), *SHELXTL* (Sheldrick, 2008 ▸) and *publCIF* (Westrip, 2010 ▸).

## supplementary crystallographic information

### catena-Poly[[[bis(pyridin-2-yl)amine]cadmium(II)]-di-µ2-azido] Crystal data

**Table:**

[Cd(N_3_)_3_(C_10_H_9_N_3_)]	*F*(000) = 720
*M**_r_* = 367.66	*D*_x_ = 1.875 Mg m^−^^3^
Monoclinic, *P*2_1_/*c*	Mo *K*α radiation, λ = 0.71073 Å
*a* = 6.6655 (5) Å	Cell parameters from 9995 reflections
*b* = 18.5106 (17) Å	θ = 3.0–32.3°
*c* = 10.5746 (9) Å	µ = 1.68 mm^−^^1^
β = 93.612 (3)°	*T* = 298 K
*V* = 1302.13 (19) Å^3^	Block, colourless
*Z* = 4	0.34 × 0.21 × 0.17 mm

### catena-Poly[[[bis(pyridin-2-yl)amine]cadmium(II)]-di-µ2-azido] Data collection

**Table:**

Bruker D8 Quest PHOTON 100 CCD diffractometer	5357 reflections with *I* > 2σ(*I*)
φ and ω scans	*R*_int_ = 0.050
Absorption correction: multi-scan (SADABS; Krause *et al.*, 2015)	θ_max_ = 36.4°, θ_min_ = 2.9°
*T*_min_ = 0.796, *T*_max_ = 0.877	*h* = −11→8
64159 measured reflections	*k* = −30→30
6322 independent reflections	*l* = −17→17

### catena-Poly[[[bis(pyridin-2-yl)amine]cadmium(II)]-di-µ2-azido] Refinement

**Table:**

Refinement on *F*^2^	Primary atom site location: dual
Least-squares matrix: full	Secondary atom site location: difference Fourier map
*R*[*F*^2^ > 2σ(*F*^2^)] = 0.055	Hydrogen site location: mixed
*wR*(*F*^2^) = 0.096	H atoms treated by a mixture of independent and constrained refinement
*S* = 1.37	*w* = 1/[σ^2^(*F*_o_^2^) + 2.1586*P*] where *P* = (*F*_o_^2^ + 2*F*_c_^2^)/3
6322 reflections	(Δ/σ)_max_ = 0.001
185 parameters	Δρ_max_ = 1.19 e Å^−^^3^
1 restraint	Δρ_min_ = −1.10 e Å^−^^3^

### catena-Poly[[[bis(pyridin-2-yl)amine]cadmium(II)]-di-µ2-azido] Special details

**Table:**

Geometry. All esds (except the esd in the dihedral angle between two l.s. planes) are estimated using the full covariance matrix. The cell esds are taken into account individually in the estimation of esds in distances, angles and torsion angles; correlations between esds in cell parameters are only used when they are defined by crystal symmetry. An approximate (isotropic) treatment of cell esds is used for estimating esds involving l.s. planes.
Refinement. Refinement of F^2^ against ALL reflections. The weighted R-factor wR and goodness of fit S are based on F^2^, conventional R-factors R are based on F, with F set to zero for negative F^2^. The threshold expression of F^2^ > 2sigma(F^2^) is used only for calculating R-factors(gt) etc. and is not relevant to the choice of reflections for refinement. R-factors based on F^2^ are statistically about twice as large as those based on F, and R- factors based on ALL data will be even larger. H-atoms attached to carbon were placed in calculated positions (C—H = 0.95 Å) and were included as riding contributions with isotropic displacement parameters 1.2 times those of the attached atoms. That attached to nitrogen was placed in a location derived from a difference map and refined with a DFIX 0.89 0.01 instruction

### catena-Poly[[[bis(pyridin-2-yl)amine]cadmium(II)]-di-µ2-azido] Fractional atomic coordinates and isotropic or equivalent isotropic displacement parameters (Å^2^)

**Table:**

	*x*	*y*	*z*	*U*_iso_*/*U*_eq_	
Cd1	0.75506 (3)	0.49321 (2)	0.57654 (2)	0.03175 (6)	
N1	0.5582 (4)	0.56986 (17)	0.4517 (3)	0.0413 (6)	
N2	0.6135 (4)	0.60430 (14)	0.3655 (3)	0.0355 (5)	
N3	0.6614 (6)	0.6371 (2)	0.2803 (3)	0.0616 (9)	
N4	0.9227 (4)	0.44425 (16)	0.4095 (2)	0.0366 (5)	
N5	0.8521 (4)	0.43719 (15)	0.3032 (2)	0.0342 (5)	
N6	0.7900 (5)	0.4295 (2)	0.2006 (3)	0.0570 (9)	
N7	0.7069 (4)	0.55560 (14)	0.7636 (2)	0.0339 (5)	
N8	0.7629 (5)	0.46074 (16)	0.9133 (2)	0.0414 (6)	
H8	0.762 (6)	0.454 (2)	0.9962 (11)	0.045 (11)*	
N9	0.8345 (4)	0.40164 (14)	0.7223 (2)	0.0323 (5)	
C4	0.6731 (6)	0.6269 (2)	0.7451 (4)	0.0472 (8)	
H4	0.656367	0.643523	0.662142	0.057*	
C5	0.6618 (7)	0.6763 (2)	0.8408 (4)	0.0566 (10)	
H5	0.638413	0.724920	0.823253	0.068*	
C6	0.6862 (6)	0.6517 (2)	0.9641 (4)	0.0553 (10)	
H6	0.679635	0.683707	1.031517	0.066*	
C7	0.7197 (5)	0.5804 (2)	0.9861 (3)	0.0447 (7)	
H7	0.735825	0.563157	1.068661	0.054*	
C8	0.7299 (4)	0.53278 (17)	0.8829 (3)	0.0329 (5)	
C9	0.7960 (4)	0.39917 (17)	0.8450 (3)	0.0332 (6)	
C10	0.7921 (7)	0.3336 (2)	0.9106 (3)	0.0513 (9)	
H10	0.764813	0.333059	0.995747	0.062*	
C11	0.8280 (8)	0.2708 (2)	0.8499 (4)	0.0619 (11)	
H11	0.823748	0.226957	0.892713	0.074*	
C12	0.8711 (7)	0.2726 (2)	0.7232 (4)	0.0566 (10)	
H12	0.898021	0.230431	0.679391	0.068*	
C13	0.8726 (6)	0.3382 (2)	0.6654 (3)	0.0467 (8)	
H13	0.902013	0.339522	0.580567	0.056*	

### catena-Poly[[[bis(pyridin-2-yl)amine]cadmium(II)]-di-µ2-azido] Atomic displacement parameters (Å^2^)

**Table:**

	*U*^11^	*U*^22^	*U*^33^	*U*^12^	*U*^13^	*U*^23^
Cd1	0.02633 (9)	0.04938 (12)	0.01945 (8)	−0.00229 (9)	0.00072 (6)	0.00442 (8)
N1	0.0259 (11)	0.0631 (18)	0.0341 (13)	−0.0045 (11)	−0.0051 (9)	0.0172 (12)
N2	0.0291 (11)	0.0394 (13)	0.0372 (13)	−0.0044 (9)	−0.0038 (9)	0.0049 (10)
N3	0.062 (2)	0.070 (2)	0.053 (2)	−0.0140 (18)	0.0035 (16)	0.0210 (17)
N4	0.0270 (11)	0.0641 (17)	0.0191 (9)	−0.0058 (11)	0.0044 (8)	−0.0026 (10)
N5	0.0303 (11)	0.0491 (14)	0.0236 (10)	−0.0058 (10)	0.0051 (8)	−0.0022 (9)
N6	0.0508 (18)	0.095 (3)	0.0247 (13)	−0.0106 (17)	−0.0029 (12)	−0.0051 (14)
N7	0.0343 (12)	0.0438 (13)	0.0242 (10)	0.0019 (10)	0.0063 (9)	0.0015 (9)
N8	0.0583 (18)	0.0509 (15)	0.0151 (10)	0.0058 (13)	0.0025 (10)	0.0018 (9)
N9	0.0336 (12)	0.0432 (13)	0.0199 (9)	0.0037 (10)	0.0003 (8)	0.0015 (8)
C4	0.051 (2)	0.0485 (19)	0.0424 (18)	0.0033 (15)	0.0094 (15)	0.0066 (14)
C5	0.064 (3)	0.0426 (19)	0.064 (3)	0.0026 (17)	0.013 (2)	−0.0035 (17)
C6	0.057 (2)	0.059 (2)	0.050 (2)	−0.0026 (18)	0.0113 (18)	−0.0211 (18)
C7	0.0459 (18)	0.060 (2)	0.0286 (14)	−0.0002 (15)	0.0059 (13)	−0.0081 (13)
C8	0.0283 (12)	0.0488 (16)	0.0219 (11)	−0.0007 (11)	0.0043 (9)	−0.0042 (10)
C9	0.0322 (13)	0.0451 (16)	0.0216 (11)	0.0021 (11)	−0.0029 (9)	0.0033 (10)
C10	0.070 (3)	0.055 (2)	0.0283 (15)	0.0042 (18)	−0.0020 (15)	0.0111 (14)
C11	0.088 (3)	0.045 (2)	0.051 (2)	0.005 (2)	−0.011 (2)	0.0118 (17)
C12	0.076 (3)	0.0447 (19)	0.047 (2)	0.0089 (18)	−0.0083 (19)	−0.0075 (16)
C13	0.058 (2)	0.0513 (19)	0.0302 (15)	0.0069 (16)	−0.0013 (14)	−0.0050 (13)

### catena-Poly[[[bis(pyridin-2-yl)amine]cadmium(II)]-di-µ2-azido] Geometric parameters (Å, º)

**Table:**

Cd1—N1	2.293 (3)	C4—C5	1.369 (5)
Cd1—N9	2.330 (2)	C4—H4	0.9300
Cd1—N7	2.330 (2)	C5—C6	1.382 (6)
Cd1—N4	2.332 (2)	C5—H5	0.9300
Cd1—N1^i^	2.394 (3)	C6—C7	1.355 (6)
Cd1—N4^ii^	2.436 (3)	C6—H6	0.9300
N1—N2	1.190 (4)	C7—C8	1.408 (4)
N2—N3	1.148 (4)	C7—H7	0.9300
N4—N5	1.199 (3)	C9—C10	1.399 (5)
N5—N6	1.145 (4)	C10—C11	1.357 (6)
N7—C8	1.330 (4)	C10—H10	0.9300
N7—C4	1.352 (4)	C11—C12	1.389 (6)
N8—C9	1.375 (4)	C11—H11	0.9300
N8—C8	1.386 (4)	C12—C13	1.361 (5)
N8—H8	0.885 (10)	C12—H12	0.9300
N9—C9	1.339 (3)	C13—H13	0.9300
N9—C13	1.351 (4)		
			
N1—Cd1—N9	158.28 (9)	C13—N9—Cd1	112.2 (2)
N1—Cd1—N7	94.44 (10)	N7—C4—C5	124.2 (4)
N9—Cd1—N7	80.59 (9)	N7—C4—H4	117.9
N1—Cd1—N4	95.09 (10)	C5—C4—H4	117.9
N9—Cd1—N4	96.82 (9)	C4—C5—C6	117.9 (4)
N7—Cd1—N4	159.02 (9)	C4—C5—H5	121.0
N1—Cd1—N1^i^	76.52 (11)	C6—C5—H5	121.0
N9—Cd1—N1^i^	83.57 (9)	C7—C6—C5	119.4 (3)
N7—Cd1—N1^i^	100.49 (10)	C7—C6—H6	120.3
N4—Cd1—N1^i^	99.89 (10)	C5—C6—H6	120.3
N1—Cd1—N4^ii^	102.19 (10)	C6—C7—C8	119.5 (3)
N9—Cd1—N4^ii^	98.22 (9)	C6—C7—H7	120.2
N7—Cd1—N4^ii^	83.17 (9)	C8—C7—H7	120.2
N4—Cd1—N4^ii^	76.57 (10)	N7—C8—N8	122.2 (3)
N1^i^—Cd1—N4^ii^	176.17 (9)	N7—C8—C7	121.8 (3)
N2—N1—Cd1	125.4 (2)	N8—C8—C7	115.9 (3)
N2—N1—Cd1^i^	118.4 (2)	N9—C9—N8	121.8 (3)
Cd1—N1—Cd1^i^	103.48 (11)	N9—C9—C10	121.4 (3)
N3—N2—N1	178.0 (3)	N8—C9—C10	116.8 (3)
N5—N4—Cd1	125.1 (2)	C11—C10—C9	120.0 (3)
N5—N4—Cd1^ii^	113.66 (19)	C11—C10—H10	120.0
Cd1—N4—Cd1^ii^	103.43 (10)	C9—C10—H10	120.0
N6—N5—N4	177.9 (4)	C10—C11—C12	119.3 (4)
C8—N7—C4	117.1 (3)	C10—C11—H11	120.4
C8—N7—Cd1	129.3 (2)	C12—C11—H11	120.4
C4—N7—Cd1	113.1 (2)	C13—C12—C11	117.6 (4)
C9—N8—C8	134.7 (2)	C13—C12—H12	121.2
C9—N8—H8	115 (3)	C11—C12—H12	121.2
C8—N8—H8	111 (2)	N9—C13—C12	124.7 (3)
C9—N9—C13	117.1 (3)	N9—C13—H13	117.7
C9—N9—Cd1	128.1 (2)	C12—C13—H13	117.7
			
C8—N7—C4—C5	0.2 (5)	C13—N9—C9—N8	177.4 (3)
Cd1—N7—C4—C5	−172.7 (3)	Cd1—N9—C9—N8	−22.4 (4)
N7—C4—C5—C6	−0.1 (6)	C13—N9—C9—C10	−1.1 (5)
C4—C5—C6—C7	−0.1 (6)	Cd1—N9—C9—C10	159.0 (3)
C5—C6—C7—C8	0.2 (6)	C8—N8—C9—N9	11.1 (6)
C4—N7—C8—N8	179.2 (3)	C8—N8—C9—C10	−170.3 (4)
Cd1—N7—C8—N8	−9.2 (4)	N9—C9—C10—C11	0.1 (6)
C4—N7—C8—C7	0.0 (4)	N8—C9—C10—C11	−178.5 (4)
Cd1—N7—C8—C7	171.5 (2)	C9—C10—C11—C12	0.9 (7)
C9—N8—C8—N7	6.0 (6)	C10—C11—C12—C13	−0.8 (7)
C9—N8—C8—C7	−174.7 (4)	C9—N9—C13—C12	1.2 (6)
C6—C7—C8—N7	−0.2 (5)	Cd1—N9—C13—C12	−162.0 (3)
C6—C7—C8—N8	−179.5 (3)	C11—C12—C13—N9	−0.2 (7)

Symmetry codes: (i) −*x*+1, −*y*+1, −*z*+1; (ii) −*x*+2, −*y*+1, −*z*+1.

### catena-Poly[[[bis(pyridin-2-yl)amine]cadmium(II)]-di-µ2-azido] Hydrogen-bond geometry (Å, º)

**Table:**

*D*—H···*A*	*D*—H	H···*A*	*D*···*A*	*D*—H···*A*
N8—H8···N6^iii^	0.89 (1)	2.21 (1)	3.086 (4)	174 (4)

Symmetry code: (iii) *x*, *y*, *z*+1.
